# Serological Diagnosis of *Brucella* Infection in Cetaceans by Rapid Serum Agglutination Test and Competitive ELISA with *Brucella abortus* and *Brucella ceti* as Antigens

**DOI:** 10.3390/pathogens14010026

**Published:** 2025-01-02

**Authors:** Tiziana Di Febo, Gabriella Di Francesco, Carla Grattarola, Luigina Sonsini, Ludovica Di Renzo, Giuseppe Lucifora, Roberto Puleio, Cristina Esmeralda Di Francesco, Camilla Smoglica, Giovanni Di Guardo, Manuela Tittarelli

**Affiliations:** 1 Istituto Zooprofilattico Sperimentale dell’Abruzzo e del Molise ‘G. Caporale’, National Reference Center for Brucellosis, 64100 Teramo, Italy; g.difrancesco@izs.it (G.D.F.); l.sonsini@izs.it (L.S.); l.direnzo@izs.it (L.D.R.); m.tittarelli@izs.it (M.T.); 2 Istituto Zooprofilattico Sperimentale del Piemonte, Liguria e Valle d’Aosta, National Reference Center for Diagnostic Investigations in Stranded Marine Mammals (C.Re.Di.Ma.), 10154 Torino, Italy; carla.grattarola@izsto.it; 3 Istituto Zooprofilattico Sperimentale del Mezzogiorno, Portici, 80055 Napoli, Italy; giuseppe.lucifora@izsmportici.it; 4 Istituto Zooprofilattico Sperimentale della Sicilia, 90129 Palermo, Italy; roberto.puleio@izssicilia.it; 5 Veterinary Medical Faculty, University of Teramo, 64100 Teramo, Italy; cedifrancesco@unite.it (C.E.D.F.); csmoglica@unite.it (C.S.); gdiguardo@unite.it (G.D.G.)

**Keywords:** *Brucella ceti*, cetaceans, competitive ELISA, Rose Bengal test, serological diagnosis

## Abstract

Rose Bengal antigen and smooth lipopolysaccharide (s-LPS) were produced from a field strain of *Brucella ceti* (“homologous” antigens) and from the reference strain *B. abortus* S99 (“heterologous” antigens); they are currently used for the diagnosis of brucellosis in cattle, water buffaloes, sheep, goats, and pigs, as recommended in the Manual of Diagnostic Tests and Vaccines for Terrestrial Animals of the World Organization for Animal Health (WOAH). “Homologous” and “heterologous” antigens were used in a rapid serum agglutination test (Rose Bengal test, RBT) and a competitive ELISA assay (c-ELISA) to test a panel of sera, blood, and other body fluids (cerebrospinal fluid, pericardial fluid, tracheal fluid, and aqueous humor) collected from 71 individuals belonging to five cetacean species (*Stenella coeruleoalba*; *Tursiops truncatus*; *Grampus griseus*; *Globicephala melas*; and *Ziphius cavirostris*), which were found stranded on the Italian coastline. Six animals were positive for *Brucella* spp. for bacterial isolation and/or PCR, and 55 animals were negative; for the remaining 10 animals, no PCR/isolation data were available. A total of 90 samples were tested. Results obtained from the two tests were compared in order to identify the most suitable antigen for the serological diagnosis of *Brucella* infection in cetaceans. The RBT performed with the “homologous” antigen showed the best results in comparison with the “heterologous” antigen: diagnostic sensitivity, specificity, and accuracy were 80.0%, 44.1%, and 46.9% for the “homologous” antigen and 80.0%, 17.0%, and 21.9% for the “heterologous” antigen. For the c-ELISA, “homologous” and “heterologous” s-LPS showed similar results (diagnostic sensitivity 66.7%, diagnostic specificity 97.3%, and diagnostic accuracy 95.0%). Therefore, the RBT using the “homologous” antigen and c-ELISA with “homologous” or “heterologous” s-LPS could be used in parallel for the detection of antibodies against *Brucella* spp. in cetaceans.

## 1. Introduction

The first reports of *Brucella* spp. infection in marine mammals date back to 1994, in pinnipeds found stranded in Scotland [1] and in a fetus of a bottlenose dolphin (*Tursiops truncatus*) beached in San Diego, California [2]. The isolates were first named as *B. maris*; after biochemical and molecular studies, two new species of *Brucella* in marine mammals were identified in 2007, named *B. ceti* (cetaceans) and *B. pinnipedialis* (pinnipeds), respectively [3]. In the Mediterranean Sea, *B. ceti* was isolated for the first time in 2012 from three specimens of striped dolphin (*Stenella coeruleoalba*), the first one of which was found stranded along the Tuscany coastline [4], while the remaining two were found on the Ionian coast of Apulia [5]. The bacterium was also isolated from striped dolphins and bottlenose dolphins in Spain and Croatia [6]. *B. ceti* infection has been confirmed either by isolation and/or PCR in many cetaceans alongside a number of pinniped species, including California sea lions (*Zalophus californianus*). Sero-epidemiological surveys and retrospective studies were conducted or are currently underway, in order to establish the prevalence of *Brucella* spp. infection in marine mammal populations worldwide [6,7,8,9,10,11,12,13,14,15,16,17].

Marine brucellae, like other “terrestrial” *Brucella* species (*B. abortus*, *B. melitensis*, *B. suis*, *B. ovis*, and *B. canis*), may cause severe subacute-to-chronic disease forms in affected animals, such as abortion, male infertility, bone and skin lesions, cardiopathies, and neurobrucellosis, leading to stranding and death. Marine brucellae are also potential zoonotic agents. In humans, *Brucella* spp. infection causes an acute febrile illness (undulant fever); the disease may also become chronic and produce serious complications affecting the cardiovascular, musculo-skeletal, and central nervous systems [14,18]. To date, *B. ceti* strains have been isolated from four human clinical cases: a laboratory worker, two individuals who regularly consumed raw fish, and a fisherman [14,19,20,21]. Although human cases are very few [14], it would be important to know the true prevalence of *Brucella* spp. infection among marine mammals, in order to protect human and marine mammals’ health, with special emphasis on that of endangered species.

Official methods for the serological diagnosis of *Brucella* spp. infection are the Rose Bengal test (RBT), complement fixation test (CFT), indirect and competitive ELISA, and fluorescence polarization assay (FPA). Recommended strains for the production of diagnostic antigens for the RBT, CFT, ELISA, and FPA are *B. abortus* strain 99 (S99) (Weybridge), *B. abortus* strain 1119-3 (S1119-3) (USDA), and *B. melitensis* strain 16M [18].

In the present study, “heterologous” antigens for the RBT and c-ELISA, produced from the reference strain *B. abortus* S99, were compared with “homologous” antigens produced from a field strain of *B. ceti*, in order to select the strain providing the best performance in the diagnosis of *Brucella* spp. infection in cetaceans. Both the RBT and c-ELISA were standardized according to the World Organization for Animal Health (WOAH) Manual [18] and to standard operative procedures of the Brucellosis European Reference Laboratory (ANSES, Maisons-Alfort, Paris, France) [22,23].

## 2. Materials and Methods

### 2.1. Ethics Statement

The present study was conducted on animals found stranded along the Italian coastline; no live animals were involved in sampling activities.

The National Reference Centre for Diagnostic Investigations on Stranded Marine Mammals (C.Re.Di.Ma.—Istituto Zooprofilattico Sperimentale del Piemonte, Liguria e Valle d’Aosta, Torino, Italy) and the Istituti Zooprofilattici Sperimentali (IIZZSS) are public laboratories authorized by the Italian Ministry of Health to perform systematic surveys on infectious diseases and, more generally, on the causes of death of marine mammals found stranded on the coast of Italy.

### 2.2. Panel of Samples and Control Sera

Samples were collected from animals found stranded lifeless along the Italian coastline (Liguria, Abruzzo, Calabria and Sicily Regions) during the period 2012–June 2023. Carcasses were submitted to the laboratories of “Istituto Zooprofilattico Sperimentale dell’Abruzzo e del Molise” (IZSAM, Teramo, Italy), “Istituto Zooprofilattico Sperimentale di Piemonte, Liguria e Valle d’Aosta” (IZSPLV—C.Re.Di.Ma., Torino, Italy), “Istituto Zooprofilattico Sperimentale del Mezzogiorno” (IZSMe, Portici, Italy), and “Istituto Zooprofilattico Sperimentale della Sicilia” (IZS Sicilia, Palermo, Italy), belonging to the network of IIZZSS laboratories, coordinated by the C.Re.Di. Ma, where a detailed post mortem examination [24], along with microbiological, parasitological, and virological analyses for the identification of agents impacting cetacean health and conservation were performed. To increase the probability of isolating *Brucella*, several culture media were used in parallel [16].

Part of these samples was stored in the collections of the University of Teramo, Faculty of Veterinary Medicine (Teramo, Italy).

A total of 90 samples (serum, blood, and other biological fluids) were collected from 71 animals (Table 1; Supplementary Tables S1 and S2).

A total of 41 sera, 11 blood samples, and 10 samples of other biological fluids (cerebrospinal fluid, aqueous humor, and pericardial fluid) were collected from 51 striped dolphins (*Stenella coeruleoalba*). A total of 6 animals out of the 51 were positive for *Brucella* (isolation and PCR), 35 animals resulted negative, and for the remaining 10 individuals, no data (PCR/isolation) were available.

A total of 13 sera, 4 blood samples, and 4 samples of other biological fluids (pericardial fluid, aqueous humor, and tracheal fluid) were collected from 16 bottlenose dolphins (*Tursiops truncatus*) and resulted negative for *Brucella* (isolation and PCR).

Moreover, a serum sample collected from a Risso’s dolphin (*Grampus griseus*); 3 samples (blood, pericardial fluid, and cerebrospinal fluid) collected from a long-finned pilot whale (*Globicephala melas*), and 3 samples (serum, blood, and cerebrospinal fluid) collected from 2 Cuvier’s beaked whales (*Ziphius cavirostris*) were analyzed. The Risso’s dolphin, the long-finned pilot whale, and the two Cuvier’s beaked whales were negative for *Brucella* (isolation/PCR).

Before testing, blood samples were centrifuged at 1500× *g* for 15 min at 4 °C to remove cellular debris, and plasma was collected.

As negative and positive controls for serological tests, a bovine serum negative for *Brucella* spp. for the RBT, CFT, c-ELISA, i-ELISA, and bacterial isolation and a bovine serum positive for *B. abortus* (2nd National Standard Serum, IZSAM, 1000 IU/mL) were used.

In order to standardize the RBT, the following sera, provided by the WOAH Reference Laboratory for Brucellosis at AHVLA Weybridge (Addlestone, Surrey, UK), were used, as recommended in WOAH Manual: WOAH Negative Standard Serum (WOAH-ELISANSS) and WOAH International Positive Standard Serum (WOAH-ISS) (titre: 1000 IU SAT, 1000 ICFTU). To standardize the c-ELISA, the WOAH Strong Positive Serum (WOAH-ELISASPSS) and Weak Positive Serum (WOAH-ELISAWPSS) were used (18, 22, 23).

### 2.3. Production of Antigens for Rose Bengal Test and ELISA

The “Homologous” and “heterologous” antigens for the RBT and purified smooth lipopolysaccharide (s-LPS) for c-ELISA were produced according to the WOAH Manual [18] using, respectively, a field strain of *B. ceti* (NRG 31957/2013 TE) isolated from the brain of a striped dolphin stranded on the southern coastline of Apulia (Otranto, Lecce, Italy) in 2013 [25] and the *B. abortus* S99 strain provided by the Central Veterinary Laboratory (CVL, Weybridge, UK).

Two batches of “homologous” s-LPS (784/2020 and 814/2020) and two batches of “heterologous” s-LPS (730/2020 and 775/2020) were produced for the coating of ELISA microplates.

### 2.4. Rose Bengal Test

The panel of samples collected from stranded Cetaceans were tested by the RBT using “homologous” and “heterologous” antigens according to the WOAH Manual: briefly, 30 µL of each sample were placed on a white plastic plate and mixed with an equal volume of RBT antigen to produce a circular zone approximately 2 cm in diameter. The mixture was incubated for 4 min at room temperature (RT) with gentle agitation using a rocker. Any agglutination reaction visible to the naked eye was considered to be positive [18].

### 2.5. Competitive ELISA

Ninety-six-well ELISA microplates (Medium Binding, Costar, Corning Inc., New York, NY, USA) were coated with 100 µL/well of “homologous” and “heterologous” s-LPS at the following dilutions in 50 mM carbonate/bicarbonate buffer, pH 9.6: 1:300 (batch 784/2020), 1:130 (batch 814/2020), 1:700 (batch 730/2020), and 1:1600 (batch 775/2020). Microplates were incubated overnight (ON) at +4 °C and blocked for 1 h at RT with 200 µL/well of 1% yeast extract (Oxoid) in PBS containing 0.05% Tween 20 (PBST). After washings with PBST, 50 µL/well of PBST (MAb Control), positive and negative bovine control sera, and samples diluted 1:10 [26] in PBST were added and incubated for 1 h at RT. Microplates were then washed 4 times with PBST, and 50 µL/well of MAb-HRP 4B5A anti-*Brucella* s-LPS (IZSAM) was added [27,28,29]. MAb-HRP was used at the following dilutions in PBST: 1:200,000 (s-LPS batches 784/2020 and 814/2020) and 1:100,000 (s-LPS batches 730/2020 and 775/2020). After incubation for 1 h at RT and further washings, 100 µL of chromogen substrate (3,3′, 5,5′-Tetramethylbenzidine, Surmodics) was dispensed into each well and incubated at RT for 30 min. The colorimetric reaction was blocked with 50 µL/well of 0.5 N sulphuric acid, and optical densities (OD450 nm) were measured at 450 nm with a microplate reader (Tecan, Switzerland). Control sera and samples were tested in duplicate. Dilutions of s-LPS and MAb-HRP were assessed by checkerboard titration.

Results were expressed as B/B0%, using the following formula:

B/B0% = [(Mean OD450 nm control sera or samples)/(Mean OD450 nm MAb Control)] × 100

The test was validated with bovine and water buffalo (*Bubalus bubalis*) positive and negative sera, according to the WOAH Manual [30] using the Receiver Operating Characteristic curves (ROC curves) [31]. A cut-off value of 50% B/B0%, giving 100% diagnostic sensitivity and specificity, was selected. This cut-off value was used in this study because, due to the low number of available true positive and negative samples, ROC curves could not be used to determine a cut-off for Cetacean species. Samples giving a B/B0% ≤ 50% were considered as positive, samples giving a B/B0% > 50% were considered as negative.

### 2.6. Data Analysis

Diagnostic sensitivity, specificity, and accuracy of RBT and c-ELISA developed with “homologous” and “heterologous” antigens, with 95% confidence interval, were estimated using the software Microsoft Excel version 2016 and 2 × 2 tables [30]. All those samples (e.g., hemolytic sera) which provided uninterpretable results were not considered in the calculation of performances.

Bacterial isolation and PCR were considered as “gold standard” tests.

## 3. Results

### 3.1. Rose Bengal Test

A total of 90 samples were tested by the RBT using both the “homologous” and the “heterologous” antigen. A total of 22 out of the 90 samples showed a high degree of hemolysis and provided non-interpretable results with both the RBT antigens.

Moreover, 40 out of the remaining 64 samples collected from animals with known health status produced agglutination when tested with the “homologous” antigen: 33 samples collected from animals that were negative for Brucella, and 4 samples collected from animals that were positive for Brucella. Furthermore, 28 samples did not produce agglutination: 26 samples collected from animals that were negative for Brucella, and 1 sample collected from an animal that was positive for Brucella.

A total of 57 out of 64 non-hemolitic samples produced agglutination when tested with the “heterologous” antigen: 49 samples collected from animals that were negative for Brucella, and 4 samples collected from animals that were positive for Brucella. Moreover, 11 samples did not produce agglutination: 10 samples collected from animals that were negative for Brucella, and 1 sample collected from an animal that was positive for Brucella.

Diagnostic specificity values for the RBT performed with “homologous” and “heterologous” antigens were, respectively, 44.1% and 17.0%; diagnostic sensitivity was 80.0% for both antigens. Results are shown in Table 2 and Table 3 as well as in Supplementary Table S1.

Regarding the four non-hemolitic samples collected from animals with unknown status, three out of four samples produced agglutination with the “homologous” antigen and four out of four samples produced agglutination when tested with the “heterologous” antigen (Supplementary Table S2).

### 3.2. Competitive ELISA

A total of 72 out of 74 Brucella negative samples were negative with both the “homologous” and “heterologous” s-LPS, and 2 resulted positive; 4 samples out of 6 Brucella-positive samples resulted positive for both “homologous” and “heterologous” s-LPS, and 2 resulted negative (Table 4).

Diagnostic specificity and sensitivity for c-ELISA performed using the two batches of “homologous” s-LPS and the two batches of “heterologous” s-LPS were similar, being, respectively, 97.3% and 66.7% (Table 4).

**Figure 1:**
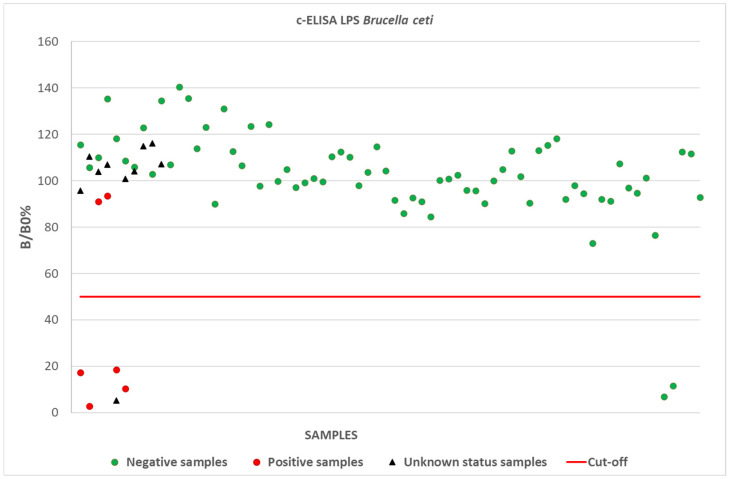
Competitive ELISA: B/B0% mean values obtained for positive, negative, and unknown status samples with *Brucella ceti* s-LPS (“homologous” antigen, batches 784/2020 and 814/2020). Similar results were obtained for *Brucella abortus* s-LPS (“heterologous” antigen, batches 730/2020 and 775/2020).

Among the 10 samples collected from animals with unknown health status, 9 resulted negative for brucellosis and 1 resulted positive with both “homologous” and “heterologous” s-LPS. ELISA results are shown in Supplementary Tables S1 and S2; B/B0% values obtained for positive, negative, and unknown status samples with the two types of s-LPS are shown in Figure 1.

## 4. Discussion and Conclusions

Brucellosis affects humans and animals (wildlife and domestic animals). In order to protect human health and prevent economic losses due to the spreading of *Brucella* spp. in farms, surveillance of brucellosis in farmed animals is regulated by national and international laws; on the contrary, brucellosis in wildlife is subjected to voluntary monitoring activities. In particular, cetaceans are migratory animals that cross jurisdictional boundaries between neighboring, coastal Nations. Many species have cyclic and predictable migration routes; migrations of other species are less predictable, so surveillance of cetacean infectious diseases is difficult [32].

Sero-epidemiological investigations aimed at detecting antibodies against *Brucella* spp. in sera of several cetacean species have been described by different authors. The diagnostic methods used are the RBT, c-ELISA, and i-ELISA [7,9,11,12,15,16,26].

The RBT is rapid, easy to perform, and sensitive and can be recommended in wildlife species as a general-purpose diagnostic test; although, positive results have to be investigated with other diagnostic methods. In domestic animals, the RBT is commonly used as a screening test due to its high sensitivity; its specificity, however, is low, so the RBT-positive results have to be confirmed by CFT. This latter test could also be used for wild animals, but the temperature of complement inactivation and cut-off values have been documented for a few species only. The disadvantage of the RBT and CFT is the requirement of high-quality serum samples to avoid uninterpretable results [18]. ELISA tests are most useful with poor quality and hemolyzed sera; these tests can also be used for testing matrices different from blood serum (plasma, other body fluids, skeletal muscle, and lung juice). Indirect ELISA requires specific secondary antibodies or proteins A and G. Anti-IgG antibodies for wild animals are not available for all the species, and those that are available have to be validated for use with other species [33,34]; the affinity of proteins A and G for IgG have been evaluated for a limited number of mammalian species [10,34]. Competitive ELISA is less sensitive than i-ELISA but is currently used to test samples collected from different species, due to the use of a single secondary antibody directed against the microplate-coating antigen; in these cases, cut-off values should be calculated for each species [30].

Many tests currently used for the diagnosis of diseases in wildlife have been developed and validated for domestic animals; validation of these tests for wild animals is difficult, due to the fact that an adequate number of serum samples collected from infected and non-infected animals would be required for each species [35].

According to the available biomedical literature, different *Brucella* smooth strains were used to produce antigens and s-LPS for the diagnosis of *Brucella* spp. infection in marine mammals: *B. abortus* 2308, *B. abortus* S19, *B. melitensis* 16M, and *B. ceti* [9]; *B. pinnipedialis* [26]; *B. abortus* [7]; *B. abortus* 125 [15]; and *B. abortus* S99 [11]. In order to harmonize the RBT, CFT, ELISA, and FPA results obtained by different laboratories, the WOAH recommended two strains of *B. abortus* (S99 and S1119-3) and one strain of *B. melitensis* (16M) for the production of diagnostic antigens and the use of WOAH-positive and -negative reference standard sera to standardize serological tests [18].

Samples for the sero-epidemiological disease surveillance in cetaceans are collected from individuals kept in aquaria/delphinaria/zoological gardens as well as from hunted animals and, in the majority of cases, from stranded cetaceans. In the latter case, samples collected from dead animals are often hemolyzed or show very poor quality (e.g., bacterial contamination or advanced post mortem autolysis): this could provide false positive or false negative results affecting the diagnostic sensitivity and specificity of serological tests.

In the present study, the performances of the RBT and c-ELISA developed using antigens produced from a field strain of *B. ceti* and from *B. abortus* S99 (reference strain) were compared, in order to select the strain providing the best results in terms of diagnostic sensitivity and specificity for the diagnosis of *Brucella* spp. infection in cetaceans, in particular in dead animals with a poor degree of post mortem preservation, alias with a more or less advanced post mortem autolysis degree [24].

The RBT performed using the “homologous” antigen showed higher values of diagnostic specificity and accuracy (respectively, 44.1% and 46.9%) in comparison to the “heterologous” antigen (17.0% and 21.9%, respectively). Diagnostic sensitivity was the same for both antigens (80%). Moreover, 22 out of the 90 samples of the panel provided non-interpretable results due to the high degree of hemolysis and could not be used in the evaluation of the diagnostic performances of the two RBT antigens. False positive results (33 out of 59 for the “homologous” antigen and 49 out of 59 for the “heterologous” antigen) could be due to the poor quality of the samples or, for some of them, to cross-reactions with bacteria similar to the brucellae that could infect cetaceans (e.g., alpha-Proteobacteria, *Yersinia enterocolitica* O:9, *Escherichia coli* O:157, and *Salmonella* spp.) (10, 18, 21); the false negative result (one out of five for both the antigens) could be due to overall low avidity or reduced titers of agglutinating antibodies [9].

Regarding the c-ELISA, the “homologous” and “heterologous” s-LPS provided similar results: for both antigens, diagnostic sensitivity, specificity, and accuracy were, respectively, 66.7%, 97.3%, and 95.0%. Moreover, no differences were reported among qualitative results obtained for the different batches of s-LPS. Two out of the six positive serum samples resulted negative for c-ELISA: one of them was collected from a striped dolphin (ID 108174/2020) negative for *Brucella* isolation but showing positivity at PCR only at the central nervous system level. The two samples also resulted negative when retested undiluted. False negative results could be due to the degradation of immunoglobulins in carcasses with advanced post mortem autolysis. The sensitivity of serological tests tends to decrease when samples are collected from animals found stranded lifeless—with neurobrucellosis representing a well-known cause of stranding and death in *B. ceti*-infected striped dolphins—in comparison to those obtained from live-stranded individuals [36,37,38,39,40]. According to the literature data, the stability of immunoglobulins could be organ/tissue-dependent and/or could vary depending on the animal species and different disease conditions [41]; samples collected post mortem from the thoracic cavity seem to be less subjected to degradation than those collected from blood vessels [36,38].

A total of 2 samples (*T. truncatus*, ID 309172/2021 and 11477/2023) out of 74 negative samples resulted positive: the first one was collected from a dolphin from which the Gram-negative bacteria *Photobacterium damselae* and *Vibrio parahaemolyticus* were isolated. Therefore, the false positive results obtained could be due to cross-reactivity with other Gram negative bacteria, antigenically similar to *Brucella*, that infect terrestrial and marine mammals [17,42,43]. The RBT results for sample ID 309172/2021 were inconclusive, due to its high degree of hemolysis; the second sample (ID 11477/2023) resulted positive with both the RBT “homologous” and “heterologous” antigens.

The health status (positivity/negativity for *Brucella* spp.) of cetaceans tested in this study was assessed using bacterial isolation and PCR as gold standards. Unfortunately, diagnosis of brucellosis could be difficult, and negative results for bacterial isolation/PCR do not exclude *Brucella* infection in serologically positive individuals. This could be due to the differential spreading of *Brucella* in organs and tissues, the degree of preservation of tissues, the bacterial load of the pathogen and competing bacteria, and other causes that could affect diagnostic results. When it occurs with living animals, they are declared “suspect cases”, isolated from other animals and placed under observation until negative serological results were obtained (false positivity) or specific symptoms of the disease are observed (true positivity). On the contrary, when it occurs with dead animals, it is difficult to know the true health status: positivity at serological tests could be due to cross-reactions (false positivity) or could be due to the presence of antibodies specific for the pathogen (true positivity).

Both the RBT and c-ELISA were standardized according to the WOAH Manual and to the Brucellosis EU RL specifications using WOAH bovine reference sera, but it was not possible to fully validate the two methods for cetaceans, due to the lack of cetacean standard sera and the low number of available true positive and negative samples.

The RBT and c-ELISA performances (diagnostic sensitivity, specificity, and accuracy) and the c-ELISA cut-off will be re-evaluated over time when additional samples will be made available.

Further investigations should also be carried out by testing serum samples collected from live animals (e.g., individuals found stranded but still alive and individuals from aquaria/delphinaria/zoological gardens) to better understand if the high rate of false positive reactions observed in the present study for the RBT, with special reference to those carried out with *B. abortus* antigen, could be due to the low quality of samples, all collected from dead animals often in a poor state of preservation, or could depend on antigenic differences between *B. ceti* and *B. abortus*. The RBT antigen is produced using heat-killed whole cells resuspended in phenol saline buffer at pH 3.65 ± 0.05 and stained with Rose Bengal stain [18]. Probably, heat-inactivation, acidification, and staining could modify the antigenic properties of *Brucella* cells, with this resulting in differences in the diagnostic performances of the two strains. Proteomics analyses could help investigate if there are differences in the protein patterns between the two strains of *Brucella* under native conditions and after chemical modifications, such as those occurring during RBT antigen production.

On the contrary, the LPS of *Brucella* smooth strains shows common epitopes, so s-LPS extracted from one strain can be used as antigen in the serological diagnosis of *Brucella* spp. infection caused by other smooth strains [18]; this homology could explain the similar results obtained in this study for c-ELISA performed with LPS produced from *B. abortus* and *B. ceti*. However, heterogeneity of the s-LPS of *Brucella* strains isolated from marine mammals, with regard to O-PS C epitope content and average size, has been described by Baucheron et al. [44]; so further studies should be conducted to better characterize the antigenic properties of s-LPSs of marine Brucellae.

The RBT and c-ELISA performed using antigens produced from *B. abortus* S99 are currently used in IZSAM Serology Laboratories for the diagnosis of brucellosis in cattle, water buffaloes, sheep, and goats (live animals). For cattle and water buffaloes, the diagnostic sensitivity and specificity of RBT are, respectively, the following: 98.1% (95% CL: 96.8–99.1%) and 96.0% (95% CL: 99.7–99.8%) [45], while for the c-ELISA described in this paper, they were both 100% (95% CL: 98.7–100.0% for sensitivity, and 99.0–100.0% for specificity). These values, with special reference to diagnostic sensitivity, are higher than those obtained in the present study for cetaceans using the same protocol, confirming that the serological diagnosis of infections using samples collected from carcasses is difficult.

In conclusion, RBT showed a better diagnostic sensitivity in comparison to c-ELISA when used to test cetacean samples; although, c-ELISA showed a higher diagnostic specificity and accuracy. RBT provided a greater number of false positive results than c-ELISA; moreover, it could not be used to test highly hemolized samples. However, its high sensitivity is very useful to detect *Brucella*-infected animals, in order to protect human health and to prevent the spreading of the disease in animal populations.

Therefore, the RBT performed using an antigen produced from *B. ceti* and c-ELISA using an s-LPS from *B. ceti* or the reference strain *B. abortus* S99, albeit showing limitations due to the quality of samples that should be tested, could be used in parallel to reveal antibodies against *Brucella* spp. in cetaceans.

The results of sero-epidemiological investigations, however, always have to be confirmed by isolation of the bacterium and by molecular methods, involving sequencing and characterization of the genome of *Brucella* strains [18], in order to evaluate the true prevalence of *Brucella* infection in cetaceans, to better assess the spread and circulation of *Brucella* spp. among marine mammal populations, and to protect the health of people and of the increasingly threatened pinnipeds and cetaceans inhabiting the seas and oceans of our Planet.

## Figures and Tables

**Table 1:** Panel of samples positive and negative for *Brucella* spp.

Species ^1^	Status (PCR/Isolation)	Matrices ^2^
*Stenella coeruleoalba* (51)	6 positive	Serum (6)
	35 negative	Serum (25), blood (11), cerebrospinal fluid (4), aqueous humor (3), pericardial fluid (3)
	not available for 10 animals	Serum (10)
*Tursiops truncatus* (16)	16 negative	Serum (13), blood (4), pericardial fluid (2), aqueous humor (1), tracheal fluid (1)
*Grampus griseus* (1)	1 negative	Serum (1)
*Globicephala melas* (1)	1 negative	Blood (1), cerebrospinal fluid (1), pericardial fluid (1)
*Ziphius cavirostris* (2)	2 negative	Serum (1), blood (1), cerebrospinal fluid (1)

^1^ In brackets: number of individuals for each species. ^2^ In brackets: number of samples analyzed for each type of matrix.

**Table 2:** Diagnostic sensitivity, specificity, accuracy, and confidence limits of the RBT performed using the “homologous” antigen. Samples with non-interpretable results (high degree of hemolysis) and samples collected from animals with unknown health status (no isolation/PCR data) were not included in the calculation.

		*Brucella* Positive (Isolation—PCR)	*Brucella* Negative (Isolation—PCR)	Total
RBT “homologous antigen”	Positives	4	33	37
	Negatives	1	26	27
	Total	5	59	64
		**Value**	**LCL (95%)**	**UCL (95%)**
Diagnostic sensitivity		80.0%	35.9%	95.7%
Diagnostic specificity		44.1%	32.1%	56.8%
Diagnostic accuracy		46.9%	35.2%	59.0%
Predictive positive value		10.8%	4.4%	24.8%
Predictive negative value		96.3%	81.7%	99.1%

**Table 3:** Diagnostic sensitivity, specificity, accuracy, and confidence limits of the RBT performed using the “heterologous” antigen. Samples with non-interpretable results (high degree of hemolysis) and samples collected from animals with unknown health status (no isolation/PCR data) were not included in the calculation.

		*Brucella* Positive (Isolation—PCR)	*Brucella* Negative (Isolation—PCR)	Total
RBT “heterologous antigen”	Positives	4	49	53
	Negatives	1	10	11
	Total	5	59	64
		**Value**	**LCL (95%)**	**UCL (95%)**
Diagnostic sensitivity		80.0%	35.9%	95.7%
Diagnostic specificity		17.0%	9.5%	28.5%
Diagnostic accuracy		21.9%	13.5%	33.5%
Predictive positive value		7.6%	3.1%	17.9%
Predictive negative value		90.9%	61.5%	97.9%

**Table 4:** Diagnostic sensitivity, specificity, accuracy, and confidence limits of the c-ELISA performed using both “homologous” and “heterologous” antigens. Samples collected from animals with unknown health status (no isolation/PCR data) were not included in the calculation.

		*Brucella* Positive (Isolation—PCR)	*Brucella* Negative (Isolation—PCR)	Total
c-ELISA	Positives	4	2	6
	Negatives	2	72	74
	Total	6	74	80
		**Value**	**LCL (95%)**	**UCL (95%)**
Diagnostic sensitivity		66.7%	29.0%	90.1%
Diagnostic specificity		97.3%	90.7%	99.2%
Diagnostic accuracy		95.0%	87.8%	98.0%
Predictive positive value		66.7%	29.0%	90.1%
Predictive negative value		97.3%	90.7%	99.2%

## Data Availability

The identified dataset can be requested via m.tittarelli@izs.it.

## Supplementary Materials

The following supporting information can be downloaded at https://www.mdpi.com/article/10.3390/pathogens14010026/s1: Table S1: Results of RBT and c-ELISA with *Brucella* negative and positive samples.; Table S2: Results of RBT and c-ELISA with samples with unknown status (isolation and PCR data not available).
